# A mutation screening of oncogenes, tumor suppressor gene *TP53* and nuclear encoded mitochondrial complex I genes in oncocytic thyroid tumors

**DOI:** 10.1186/s12885-015-1122-3

**Published:** 2015-03-21

**Authors:** Cecilia Evangelisti, Dario de Biase, Ivana Kurelac, Claudio Ceccarelli, Holger Prokisch, Thomas Meitinger, Paola Caria, Roberta Vanni, Giovanni Romeo, Giovanni Tallini, Giuseppe Gasparre, Elena Bonora

**Affiliations:** 1 Department of Medical and Surgical Sciences (DIMEC), Policlinico S. Orsola-Malpighi, Unit of Medical Genetics, University of Bologna, Bologna, Italy; 2 Department of Biomedical and Neuromotor Sciences (DIBINEM), Cell Signaling Laboratory, University of Bologna, Bologna, Italy; 3 Department of Diagnostic, Experimental and Specialty Medicine (DIMES), Unit of Anatomic Pathology, Bellaria Hospital, University of Bologna, Bologna, Italy; 4 Department of Diagnostic, Experimental and Specialty Medicine (DIMES), Unit of Anatomy, Policlinico S. Orsola-Malpighi, University of Bologna, Bologna, Italy; 5 Helmholtz Zentrum München Deutsches Forschungszentrum für Gesundheit und Umwelt, Neuherberg, Germany; 6 Department of Biomedical Sciences, University of Cagliari, Cagliari, Italy

**Keywords:** Oncocytic carcinoma, Nuclear mitochondrial complex I subunits, Oncogene mutation analysis

## Abstract

**Background:**

Thyroid neoplasias with oncocytic features represent a specific phenotype in non-medullary thyroid cancer, reflecting the unique biological phenomenon of mitochondrial hyperplasia in the cytoplasm. Oncocytic thyroid cells are characterized by a prominent eosinophilia (or oxyphilia) caused by mitochondrial abundance. Although disruptive mutations in the mitochondrial DNA (mtDNA) are the most significant hallmark of such tumors, oncocytomas may be envisioned as heterogeneous neoplasms, characterized by multiple nuclear and mitochondrial gene lesions. We investigated the nuclear mutational profile of oncocytic tumors to pinpoint the mutations that may trigger the early oncogenic hit.

**Methods:**

Total DNA was extracted from paraffin-embedded tissues from 45 biopsies of oncocytic tumors. High-resolution melting was used for mutation screening of mitochondrial complex I subunits genes. Specific nuclear rearrangements were investigated by RT-PCR (RET/PTC) or on isolated nuclei by interphase FISH (PAX8/PPARγ). Recurrent point mutations were analyzed by direct sequencing.

**Results:**

In our oncocytic tumor samples, we identified rare *TP53* mutations. The series of analyzed cases did not include poorly- or undifferentiated thyroid carcinomas, and none of the TP53 mutated cases had significant mitotic activity or high-grade features. Thus, the presence of disruptive *TP53* mutations was completely unexpected. In addition, novel mutations in nuclear-encoded complex I genes were identified.

**Conclusions:**

These findings suggest that nuclear genetic lesions altering the bioenergetics competence of thyroid cells may give rise to an aberrant mitochondria-centered compensatory mechanism and ultimately to the oncocytic phenotype.

**Electronic supplementary material:**

The online version of this article (doi:10.1186/s12885-015-1122-3) contains supplementary material, which is available to authorized users.

## Background

Non-medullary thyroid carcinoma (NMTC) is a well-differentiated thyroid cancer of follicular cell origin, either papillary thyroid carcinoma (PTC) or follicular thyroid carcinoma (FTC), which represents the most common endocrine malignancy. The annual incidence rate throughout the world ranges from 0.5 to 10 cases per 100,000 individuals with a two- to four-fold higher incidence of new thyroid cancer cases in women than in men [1]. The major known environmental risk factor for PTC, which represents about 80% of all thyroid cancers, is a prior exposure to radiation, with a dose-dependent effect on cancer risk. Other risk factors include iodine deficiency and excess, previous history of benign/autoimmune thyroid disease, as well as a positive family history [2].

A specific sub-phenotype in NMTC is represented by thyroid tumors with oncocytic features, which reflects the unique biological phenomenon of mitochondrial hyperplasia in the cytoplasm of oncocytic cells, characterized by their prominent eosinophilia (or oxyphilia) caused by mitochondrial abundance, from where the histopathological feature of swollen (oncòs) cells originate. For a thyroid cancer to be diagnosed as oncocytic, at least 75% of neoplastic cells ought to display the typical mitochondrial hyperplasia according to the 2004 World Health Organization classification [3].

Thyroid oncocytic tumors (with the exception of the rare oncocytic variant of medullary carcinoma) originate from follicular cells. They can be benign (oncocytic adenomas) or malignant (oncocytic carcinomas). It is generally accepted that oncocytic tumors in the thyroid and in other organs alike should be considered as distinct subtypes, since their features are peculiar enough to set them apart from corresponding neoplasms lacking accumulation of mitochondria (World Health Organization, 2004). Accordingly, oncocytic thyroid carcinomas are now classified as variants of follicular carcinoma (commonly) or of papillary carcinoma (less commonly). Interestingly, oncocytic carcinoma (OC) have long been considered a more aggressive subtype than PTC or FTC, particularly since they often appear to be refractory to radioactive iodine treatment and have poor chemo-sensitivity [4]. Overall, canonical histopathological criteria such as invasion of the tumor capsule or blood vessels are considered in order to distinguish benign versus malignant forms, regardless of the occurrence of an oncocytic phenotype. Among the molecular hallmarks of this phenotype, it has to be underlined that, in keeping with the observation that most of the time oncocytic cells mitochondria display a deranged morphology and function [5], disruptive mutations in the mitochondrial DNA (mtDNA) are nowadays univocally considered as the most prominent and frequent genetic signature for oncocytic tumors of the thyroid and other organs as well [6].

We and other groups have thereby demonstrated that pathogenic mutations in mtDNA encoded-genes impairing complex I are genetic markers of thyroid oncocytic tumors [7-9], albeit it has to be noted that in other organs, such as kidney and pituitary gland, the correlation between the occurrence of such mutations, the oncocytic phenotype and the functional disruption of complex I activity is far more stringent than in the thyroid [6,10-13]. Thyroid tumors may present as heterogeneous neoplasms, in which oncocytic cells are more or less a predominant component, and heterogeneity of nuclear and mitochondrial gene lesions may be envisioned [5,14]. Overall, since oncocytic features are present both in PTC and FTC and a number of oncocytic thyroid cancers are devoid of mtDNA disruptive mutations [7], the nuclear profile of oncocytic thyroid tumors is worth investigating, in order to pinpoint the mutations that may trigger the first hit in thyroid oncogenesis and may help in distinguishing, together with histological and cytological data, oncocytic tumors subtypes.

In forty-five oncocytic tumors of known mitochondrial DNA mutation status we therefore performed a screening survey of the nuclear encoded subunits of mitochondrial complex I, and of genes typically altered in thyroid-specific tumors such as *B-Raf proto-oncogene* (*BRAF), Harvey rat sarcoma viral oncogene homolog (H-RAS)*, *Neuroblastoma RAS viral oncogene homolog (N-RAS)*, and *Kirsten rat sarcoma viral oncogene homolog (K-RAS)*, the fusion genes *REarranged during Transfection (RET)/PTC1*, *RET/PTC3*, *Paired Box 8* (*PAX8)*/*peroxisome proliferator-activated receptor gamma (PPARγ)*, and *Tumor Protein p53 (TP53)*.

## Methods

### Tissue samples features

Forty-five tumor tissues samples were obtained from the Department of Experimental, Diagnostic and Specialty Medicine (DIMES), University of Bologna. Clinical and histological characterization was performed as previously described [7]. Briefly, 16 were hyperplastic oncocytic thyroid nodules, 7 were thyroid follicular adenomas (FA) and 22 were oncocytic thyroid carcinomas. Average patient age was 53 for patients with oncocytic lesions. All tumors were sporadic. The study was approved by the Ethical Committee of Azienda Ospedaliero-Universitaria of Bologna, protocol number 26/2009/U/Tess and handling of samples and clinical data proceeded accordingly. Patients’ description is reported in Additional file 1: Table S1. Written informed consent was obtained for each patient included in the study and all data from the patients were handled in accordance with the local ethical committee approved protocols and in compliance with the Helsinki declaration.

### Screening of TP53, BRAF, H-RAS, K-RAS and N-RAS genes

All thyroid oncocytic samples were screened for *TP53* mutations by polymerase chain reaction (PCR) and direct sequencing, as reported before [15]. PCR products were purified onto Millipore PCR clean-up plates, resuspended in bi-distilled water, and directly sequenced on both strands using BigDye v1.1 (Life Technologies) according to manufacturer’s instructions. Samples were loaded on an ABI3730 automated sequencing machines (Life Technologies) and analyzed using Sequencer v2.1.

Detection of *BRAF* p.600 V > E and *RAS* codon 61 mutations was performed using PCR primers as reported in [16], sequenced using a CEQ2000 Genetic Analysis Systems (Beckman Coulter, Fullerton, CA, USA) and analyzed using CEQanalyzer software (Beckman Coulter, Fullerton, CA, USA) as previously described [16].

### RET/PTC analysis

Total RNA was extracted using the RecoverAll kit (Ambion Inc., Austin, Texas, USA) starting from four 20-μm-thick slides, in accordance to the manufacturer’s instructions. RNA concentration was measured using Quant-it^TM^ RNA kit (Invitrogen, Carlsbad, California). Reverse-transcription PCR was performed using the Transcriptor High Fidelity cDNA Synthesis Sample Kit (Roche Diagnostic, Mannheim, Germany) and cDNA amplified using the FastStartTaq DNA polymerase reagents (Roche Applied Science, Mannheim, Germany), starting from about 100 ng of extracted RNA. *RET* rearrangement was analyzed by real time RT-PCR using primers specific for *c-RET* exons 10–11, *c-RET* exons 12–13, *RET/PTC1* and *RET/PTC3* as previously described [17]. Real time RT-PCR reactions were run in duplicate. The beta-Actin reference gene was used as RNA control. Real-time PCR was performed using an ABI SDS 7000™ instrument (Applied Biosystems, Foster City, CA, USA).

### *PAX8/PPARγ* analysis

**Figure 1:**
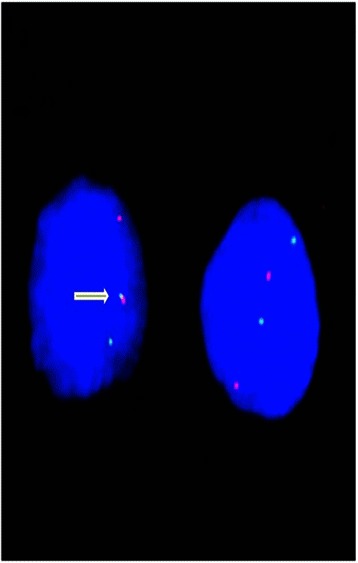
***PAX8/PPARγ*rearrangement observed in isolated nuclei from an oncocytic tumor biopsy.** The white arrow indicates the gene fusion observed with the two differently labeled probes. See text for details.

To identify the *PAX8/PPARγ* rearrangement, a dual-color single-fusion home-brew probe containing BACs RP11-339 F22 (for *PAX8*) labeled with Spectrum Orange (Abbott Molecular/Vysis Downers Grove, IL) and RP11-167 M22 (for *PPARγ*) labeled with Spectrum Green (Abbott Molecular/Vysis) was designed. Cytogenetic and fluorescence *in situ* hybridization (FISH) studies were performed as described [18]. Evaluation of the results was done by counting 25–105 nuclei (mean 65) per case, depending on the quality of preparations, using a digital image analysis system based on an epifluorescence Olympus BX41 microscope and charge-coupled device camera (Cohu), interfaced with the CytoVysion system (software 2.81 Applied Imaging, Pittsburg, PA, USA). Normal nuclei were identified by two orange and two green FISH signals, nuclei with *PAX8/PPARγ* gene fusion were identified by one orange, one green and one fused orange/green signal. An example of the observed nuclear pattern is reported in Figure 1.

### Mutation screening of nuclear mitochondrial complex I subunits

Total DNA was extracted from tissues by the use of NucleoSpin Tissue extraction kit (Machery-Nagel) according to the manufacturer’s instructions. All DNAs were pre-amplified using the GenomiPhi Illustra v2.0 amplification kit starting from 10 ng genomic DNA from tumor tissues according to the manufacturer’s instructions (GE Healthcare, UK). A screening analysis for mutations in the nuclear subunits of mitochondrial complex I and assembly factors for complex I was carried out by high resolution melting point analysis (HRMA, Idaho Technology, USA) of PCR products of the coding and flanking intronic regions of these genes from pre-amplified DNA as described [19,20].

All PCR products presenting an aberrant melting profile were re-amplified from the corresponding original genomic DNA with the same PCR primers included in the screening. Sequence analysis (ABI3730, Life Technologies) was performed according to the manufacturer. PCR primer sequences and conditions were performed as reported [19,20].

## Results

### Nuclear mitochondrial complex I mutation screening

The DNA extracted from 45 sporadic thyroid oncocytic tumors was screened for mutations in the 38 nuclear genes encoding the subunits of mitochondrial complex I and two known complex I assembly factors (ECSIT; C6orf66). For all tumor samples the mtDNA mutation status has been previously determined [7]. We identified four heterozygous changes in four complex I genes. Two of these were variants already present in public databases: the missense change p. 81Arg > Gln in *NDUFB1* (NM_004545.3, c.242G > A, rs72691104), with a very low frequency in control population (A = 1, G = 8599; m.a.f. = 0.0116, http://evs.gs.washington.edu/EVS/) and a common silent change in *NDUFC2*, corresponding to dbSNP ID rs534418 (m.a.f = 0.8546). Furthermore, an in-frame deletion of one amino acid residue was identified, c.398_400del3 p.(K133_I134delinsI) in *NDUFA12*, which despite having no dbSNP entry, was found in the control population at a low frequency (Table 1). Moreover, a novel variant was detected, namely the missense change p.8Glu > Val in *NDUFB6*, in an oncocytic carcinoma. The change was absent from 400 control chromosomes (blood-derived DNA) and from public databases (1000 Genomes and NIH-Exome Variant Server). *In silico* prediction of the putative functional effect was carried out with the programs PolyPhen-2, Provean and SIFT, all of which indicated as damaging the variant p.8Glu > Val in *NDUFB6*, whereas conflicting results arose for the p. 81Arg > Gln in *NDUFB12* (Table 1 and Additional file 1: Table S2). This sample also carried the mtDNA m.11403G > A, inserting a premature stop-codon in ND4 (p.W215Ter, Additional file 1: Table S1).

**Table 1:** **Coding variants identified in nuclear mitochondrial complex I genes. Het = heterozygotes**

Gene	Position in cDNA	Number of het oncocytic Thyroid	Number of het in EVS^a^	Type of change	PolyPhen-2 score (HumVar)
*NDUFA12*	c. 398–400 del_AGA (NM_018838.4)	1/45	21/6259	p.133del (Lys_Ile134insIle)	---
*NDUFB1*rs72691104	c. 242G > A (NM_004545.3)	1/45	1/4300	p.Arg81Gln	0.890
*NDUFB6*	c. 125A > T (NM_002493.4)	1/45	---	p.Glu8Val	0.852

^a^EVS (http://evs.gs.washington.edu/EVS/) accession as by June, 26^th^ 2014.

The *NDUFB6* affecting p.8 Glu residue maps to the mitochondrial targeting sequence (MTS) of the protein, in a position highly conserved throughout species (Additional file 2: Figure S1A); therefore, it is reasonable to hypothesize that such a non-conservative change may be highly deleterious for the correct mitochondrial localization of the protein. In addition, this change resulted to be tumor-specific (Additional file 2: Figure S1B). The one-amino acid deletion in *NDUFA12* was instead present also in the non-cancer tissue surrounding the lesion. This case also carried the *PAX8/PPARγ* rearrangement (see below).

### Evaluation of mutations in BRAF and RAS, and RET/PTC1-3 and PAX8-PPARγ rearrangements

The *BRAF*^V600E^ mutation was found in 2/45 samples (4.4%); *RAS* genes (*H-RAS*, *K-RAS* and *N-RAS*) were collectively mutated in 3/45 samples (6.7%). The *RET/PTC1* rearrangement was analyzed in 26 cases and it was found in 1 out of 26 (3.8%). Results are presented in Table 2.

**Table 2:** **Oncogenes altered in oncocytic thyroid tumors**

Oncogene	Type of mutation	Type of change	Number of oncogenic events^a^
*RET/PTC*	rearrangement	RET/PTC1	1/26
*PAX8-PPARγ*	rearrangement	---	5/10
*RAS* (*H-RAS*, *K-RAS*, and *N-RAS*)	point mutation	p.61 Gln > Arg (Q61R *H-*, *N-*, and *K-RAS*)	3/45
*BRAF*	point mutation	p.600Val > Glu (V600E)	2/45

^a^Total numbers of tested samples are different, since the different analyses were not possible in all tissues.

Considering the high frequency of mtDNA mutations in these samples and the role of PPARγ in mitochondrial biogenesis [21,22], we next hypothesized that *PPARγ* rearrangement might be preferentially associated with occurrence of mtDNA mutations. As a pilot study, *PAX8-PPARγ* rearrangements were analyzed in 10 samples, previously characterized for mtDNA mutations, in order to investigate whether this event is an alternative or concurrent mutational hit with mtDNA mutations in oncocytic thyroid lesions. The *PAX8-PPARγ* rearrangement was found in 5 out of 10 cases (mean fusion: 12.46%). Three samples carried concurrently the rearrangement and mtDNA mutations, and three samples negative for the rearrangement carried mtDNA mutations (Additional file 1: Table S1). These findings suggest the lack of a stringent association between *PAX8-PPARγ* and mtDNA mutations.

### TP53 mutation screening

All 45 tumor samples were screened for *TP53* mutations: we identified two frameshift deletions and one missense change in 3 cases (6.7%; Table 3). All changes were detected as heterozygous variants. The missense change p.364 Ala > Thr was present in a sample from an oncocytic carcinoma, carrying also a frameshift mutation in mtDNA-encoded ND4 subunit (m.11038delA). The *TP53* missense change was not present in databases of controls (i.e. ESP), but it has been reported in COSMIC as somatic mutation in ovarian cancer (accession n: COSM46361). Different prediction programs gave discrepant results on its pathogenicity (Additional file 1: Table S2).

**Table 3:** **Mutations in** ***TP53*** **tumor suppressor gene**

Base change (NM_000546)	Amino acid change	Number of het samples
c. 728delC	frameshift	1/45
c. 1248delC	frameshift	1/45
c. 1341G > A	missense change (p.364Ala > Thr)	1/45

One heterozygous deletion at c.728 was present in a sample from an oncocytic carcinoma, carrying the m.10885del, inserting a stop codon at amino acid 61 in mtDNA-encoded subunit ND4. The frameshift c.1248del was present in one sample, from an oncocytic adenoma, carrying also the *N-RAS* mutation. None of the *TP53* mutated cases had poorly- or undifferentiated histologic features.

The (co)occurrence of all genetic lesions identified is reported in Additional file 1: Table S1.

## Discussion

Previous work carried out by our group has shed light on the tight correlation between the co-occurrence of mtDNA alterations, the oncocytic phenotype, and a heavy dysfunction in the oxidative phosphorylation (OXPHOS) complexes activity, in particular in complex I [4,13]. The strength of a correlation between mtDNA mutations and functional impairment of complex I is even more striking in other oncocytomas, e.g. renal and pituitary oncocytic tumors [11-14].

Oncocytic thyroid tumors of follicular cell derivation are classified by the World Health Organization 2004 as distinctive histologic variants of FTC and PTC. This would suggest that they carry genetic abnormalities similar to those of their corresponding non-oncocytic counterparts (FTC and PTC) [23].

In comparison with other tumor types comprehensive molecular analyses of oncoytic thyroid tumors, including comparative genomic hybridization studies, have not been widely reported [24]. Recent work on oncocytic thyroid carcinomas found a series of recurrent deletion/amplification in different chromosomes [25], confirming previously reported associations with chromosome instability [26]. This level of chromosome instability is remarkable when compared with that of other types of non-oncocytic differentiated thyroid cancer. *BRAF* mutations, *RET/PTC* or *PAX8-PPARγ* rearrangements were not identified [25]. *RAS* mutations were found in oncocytic FTCs with a much lower prevalence compared to the one of the corresponding non-oncocytic FTCs [25].

The finding of chromosome instability in oncocytic thyroid carcinoma may contribute to explain the peculiar phenotype of the tumors, i.e. the aberrant mitochondrial hyperplasia that, in a relatively high percentage of cases, is tightly associated to the occurrence of clearly pathogenic mitochondrial DNA mutations.

A complete analysis of the genomic landscape of oncocytic thyroid tumors, correlated with the co-occurrence of mtDNA mutations and in other complex I nuclear-encoded genes, has not been reported so far. Therefore, we performed an extensive mutation analysis of oncocytic tumor biopsies, previously characterized for the presence of mtDNA mutations. The presence of the best-known oncogenic events in thyroid cancer, including *BRAF*, *RAS*, *TP53* mutations, *RET/PTC* and *PAX8/PPARγ* rearrangements, was assessed in addition to a high-throughput mutation screening for the nuclear-encoded complex I subunits [27], which may account for those cases lacking mtDNA mutations. The resulting data show that, in our samples, the *BRAF*, *RAS*, *RET/PTC* oncogenic events are relatively rare, similar to what observed by Ganly et al. [25]. On the other hand, the *PAX8/PPARγ* rearrangement did not show any significant correlation with the presence of mtDNA mutations, although the analysis was performed as a pilot study on a small number cases, which may also explain the relatively high frequency of *PAX8/PPARγ* rearrangement with respect to previously published data.

Our study shows that heterozygous *TP53* disruptive mutations are present in a small subset of oncocytic tumors. Two cases were oncocytic follicular carcinomas and one was diagnosed as oncocytic follicular adenoma. *TP53* mutations are typically associated with poorly differentiated and anaplastic thyroid carcinoma [28]. The series of cases that we analyzed did not include poorly- or undifferentiated thyroid carcinomas, and none of our TP53 mutated cases had significant mitotic activity or high-grade features. Thus, the presence of disruptive *TP53* mutations, albeit in a subset of cases, was completely unexpected. Interestingly, *TP53* mutations have been recently reported in 4 of 18 oncocytic carcinomas using Targeted Next-Generation Sequencing [29].

The occurrence of *TP53* mutations in oncocytic tumors that do not carry the features of poorly-differentiated or anaplastic thyroid cancers is intriguing. Two of our *TP53* mutated samples also harboured mtDNA mutations. Tumor suppressor p53 has been largely implicated in the metabolic remodeling that cancer cells develop during progression, particularly through the regulation of mitochondrial respiration via TIGAR and COXIV of the respiratory chain [30]. Nevertheless, several studies have shown that in thyroid oncocytic tumors a burden of mtDNA mutations all impinging on the bioenergetics competence of thyroid cells may give rise to an aberrant mitochondria-centered compensatory mechanisms and ultimately to the oncocytic phenotype [14].

In contrast to the findings that disruptive mtDNA mutations, in particular in genes encoding complex I subunits, are fairly common in oncocytic tumors, we did not identify a large number of nuclear-encoded complex I genetic abnormalities, suggesting that mutations in these genes do not play a major role in oncocytic thyroid cancer. This indicates that other genomic alterations may induce metabolic microenvironment changes drivers of tumorigenesis, coupled to mitochondrial abnormalities [13,31].

## Conclusions

Characterizing the genomic landscape both at nuclear and mitochondrial levels in oncocytic thyroid tumors reveals a complex genetic interplay that may also confer prognostic differences. Available massive sequencing technologies, leading to the simultaneous analysis of hundreds of different genetic regions, are increasing the molecular characterization of solid tumors. Based on our data that show the co-occurrence of multiple genetic damages, a similar approach is indicated also for the characterization of oncocytic thyroid tumors, in order to identify the best therapeutic targets for a personalized treatment of thyroid cancer subtypes.

## Additional files

Additional file 1: Table S1. Clinical characteristics and molecular defects nuclear genes and mtDNA alterations. **Table S2**. PROVEAN and SIFT output for the rare variants identified in oncocytic tumors.
Additional file 2: Figure S1. (A) Protein sequence alignment showing the conservation across species of NDUFB6 p.8 Glu. (B) Electropherograms showing the novel missense change in NDFUB6 p.8 Glu > Val. Tumor tissue sample showing a somatic heterozygous profile, compared to perilesional tissue. The two different alleles were distinguished by cloning the PCR products into pcDNAII vector and sequencing the different clones.

## Abbreviations

NMTC: Non-medullary thyroid carcinoma
PTC: Papillary thyroid carcinoma
FTC: Follicular thyroid carcinoma
OC: Oncocytic carcinoma
mtDNA: Mitochondrial DNA (mtDNA)
FA: Follicular adenoma
