# Supplementary material for: A mutation screening of oncogenes, tumor suppressor gene TP53 and nuclear encoded mitochondrial complex I genes in oncocytic thyroid tumors
Source: BMC Cancer. 2015 Mar 21;15:157. doi: 10.1186/s12885-015-1122-3 (PMC4374372; doi:10.1186/s12885-015-1122-3)
Supplement: Additional file 1: Table S1. — Clinical characteristics and molecular defects nuclear genes and mtDNA alterations. Table S2. PROVEAN and SIFT output for the rare variants identified in oncocytic tumors. [file 12885_2015_1122_MOESM1_ESM.docx]

**Supplementary Table 1:** Clinical characteristics and molecular defects nuclear genes and mtDNA alterations

| **ID code^a^** | **Classification** | **Histological description^b^** | **RET/PTC** | **Mutations in Oncogenes (H,K, N-RAS, BAF)** | **PAX8 PPARg** | **mtDNA mutations^a^** | **Type of aa change^a^** | **Complex I subunit gene^a^** | **Heteroplasmy** | **Mutations inTP53** | **Mutations in nuclear-encoded complex I genes** |
| --- | --- | --- | --- | --- | --- | --- | --- | --- | --- | --- | --- |
| HCT 01 | oncocytic hyperplasia | Diffuse and nodular thyroid hyperplasia, with large oxyphilic metaplasia | wt | wt | na | **m.13414G>A** | G360* | ND5 | + | wt | wt |
| HCT 02 | oncocytic encapsulated PTC | fvPTC, two areas of encapsulated PTC in isthmus, in right lobe fvPTC variant with oxyphilic cells, each of 0.7 cm in size, in multinodular hyperplastic area | wt | wt | **PAX8/PPARγ fusion (13.3%)** | wt | wt | wt |  | wt | wt |
| HCT 03 | oncocytic hyperplasia | Multinodular hyperplasia of left nodule, with oncocytic feature | wt | wt | na | wt | wt | wt |  | wt | wt |
| HCT 04 | oncocytic hyperplasia | Major oxyphilic hyperplastic nodule, and minor nodules present in Hashimoto thyroiditis | wt | wt | na | **m.5185G>A** | W239* | ND2 | - | wt | wt |
| HCT 05 | oncocytic hyperplasia | Oxyphilic hyperplastic nodule present in Hashimoto thyroiditis | wt | wt | na | wt | wt | wt |  | wt | wt |
| HCT 06 | oncocytic follicular carcinoma | −−− | na | wt | na | **m.1567T>4C** | S310P | CYTB | - | wt | wt |
| HCT 07 | oncocytic hyperplasia | Multinodular goiter with oncocytic metaplasia. No Hashimoto | wt | wt | na | **m.13870A>T** | K512* | ND5 | + | wt | wt |
| HCT 08 | oncocytic hyperplasia | Nodules of different size. MicroPTC oncocytic 3 mm diameter (left lobe) | wt | wt | na | wt | wt | wt |  | wt | wt |
| HCT 09 | oncocytic hyperplasia | Nodular hyperplasia, in follicular lymphocitic thyroiditis, with multifocal oncocytic metaplasia | na | wt | na | **m.11613T>C** | L285P | ND4 | - | wt | wt |
| HCT 11 | oncocytic hyperplasia | Hyperplastic thyroid nodule, with focal oncocytic features. Lymphocitic thyroiditis | na | wt | na | wt | wt | wt |  | wt | wt |
| HCT 12 | oncocytic hyperplasia | Hyperplastic multinodular goiter with colloido-cystic features. Diffuse lymphocytic thyroiditis | na | wt | na | wt | wt | wt |  | wt | wt |
| HCT 13 | oncocytic hyperplasia | Graves-Flaiani-Basedow disease with diffuse nodules;oncocytic microPTC in right lobe (4 mm diameter). | na | wt | na | wt | wt | wt |  | wt | wt |
| HCT 14 | oncocytic follicular carcinoma | −−− | na | wt | na | wt | wt | wt |  | wt | wt |
| HCT 15 | oncocytic papillary carcinoma | −−− | wt | **BRAF p.V600E** | na | wt | wt | wt |  | wt | wt |
| HCT 16 | oncocytic papillary carcinoma | Oncocytic carcinoma, 11 mm diameter, initially invasive. Hyperplastic nodule micro-macrofollicular 14 mm with micro-fvPTC | wt | wt | na | **m.3331del242bp** | fr.shift | ND1 | + | wt | wt |
| HCT 17 | oncocytic follicular carcinoma | Multinodular goiter, oncocytic follicular carcinoma minimally invasive | wt | **NRAS p.Q61R** | na | wt | wt | wt |  | **c.1248 delC, p.Arg133fs** | wt |
| HCT 18 | oncocytic papillary carcinoma | In right lobe oncocytic fvPTC, with psammoma bodies (28 mm diameter) | wt | wt | no fusion | G4975A | G169E | ND2 | - | wt | wt |
| HCT 19 | oncocytic papillary carcinoma | Warthin-like oncocytic PTC | **RET/PTC1** | wt | na | wt | wt | wt |  | wt | wt |
| HCT 20 | oncocytic follicular carcinoma | −−− | na | wt | na | wt | wt | wt |  | wt | wt |
| HCT 21 | oncocytic hyperplasia | Multinodular hyperplastic goiter, oncocytic and colloido-cystic features | wt | wt | na | **m.3571insC** | AA101* | ND1 | + | wt | wt |
| HCT 22 | oncocytic follicular carcinoma | −−− | na | wt | na | wt | wt | wt |  | wt | wt |
| HCT 23 | oncocytic hyperplasia | Multinodular oncocytic goiter. Micro-fvPTC, 2 mm and 1 mm diameter. Presence of microlipoma | wt | wt | na | **m.10537G>A** | G35E | ND4L | - | wt | wt |
| HCT 24 | oncocytic follicular adenoma | −−− | wt | wt | no fusion | wt | wt | wt |  | wt | wt |
| HCT 25 | oncocytic hyperplasia; Hashimoto's thyroditis | −−− | wt | wt | **PAX8/PPARγ fusion (3.0%)** | **m.12056G>A** | E433K | ND4 | - | wt | wt |
| HCT 26 | oncocytic follicular carcinoma | −−− | na | wt | na | **m.3571insC** | AA101* | ND1 | - | wt | wt |
| HCT 27 | oncocytic follicular carcinoma | −−− | na | wt | na | **m.11084delCA** | AA113* | ND4 | - | wt | wt |
| HCT 28 | oncocytic follicular carcinoma minimally invasive | In left lobe minimally invasive oncocytic follicular carcinoma 4 mm diameter | wt | wt | no fusion | **m.11038delA** | AA99* | ND4 | - | **c.1341G>A, p.Ala364Thr** | wt |
| HCT 29 | oncocytic follicular carcinoma minimally invasive | −−− | wt | wt | na | **m.13235insT** | AA311* | ND5 | - | wt | wt |
| HCT 30 | oncocytic follicular adenoma | Capsulated oncocytic adenoma in right lobe | na | wt | **PAX8/PPARγ fusion (20.0%)** | **m.15209T>C** | Y155H | CYTB | - | wt | wt |
| HCT 31 | oncocytic papillary carcinoma (follicular variant) encapsulated, 0.7 cm size | fvPTC with oncocytic features, 7 mm diameter | na | **HRAS p.Q61R** | na | **m.8839G>A** | A105T | ATP6 | - | wt | wt |
| HCT 32 | oncocytic follicular adenoma | −−− | na | wt | **PAX8/PPARγ fusion (8.0%)** | wt | wt | wt |  | wt | wt |
| HCT 33 | minimally invasive follicular oncocytic carcinoma | −−− | na | wt | **PAX8/PPARγ fusion (18.0%)** | **m.4720G>A** | W84* | ND2 | - | wt | wt |
| HCT 34 | oncocytic hyperplasia; Hashimoto's thyroditis | −−− | wt | wt | no fusion | wt | wt | wt |  | wt | wt |
| HCT 35 | oncocytic hyperplasia; Hashimoto's thyroditis | −−− | wt | wt | na | wt | wt | wt |  | wt | wt |
| HCT 36 | oncocytic papillary carcinoma | In left lobe PTC classic and follicular variant, oncocytic features in multinodular colloido-cystic goiter | wt | wt | na | **m.11475G>A** | G239D | ND4 | - | wt | wt |
| HCT 37 | oncocytic follicular adenoma | Multiple foci of fvPTC in hyperplastic nodule (the biggest one 6 mm diameter). No invasivity | wt | **KRAS p.Q61R** | na | **m.3949T>C** | Y215H | ND1 | - | wt | wt |
| HCT 38 | oncocytic follicular adenoma | Oncocytic lesion in right lobe with flogistic and xanthomatous features. No invasivity | wt | wt | na | **m.11403G>A** | W215* | ND4 | - | wt | **NDUFB1 rs72691104; p.Arg81Gln** |
| HCT 39 | follicular oncocytic carcinoma | Widely invasive oncocytic follicular carcinoma | wt | wt | na | **m.3392G>A** | G29D | ND1 | - | wt | wt |
| HCT 40 | oncocytic follicular adenoma | Lesional diameter 3 cm | wt | wt | no fusion | **m.13042G>A** | A236T | ND5 | + | wt | wt |
| HCT 41 | oncocytic papillary carcinoma | Tall cells variant | wt | **BRAF p.V600E** | na | wt | wt | wt |  | wt | wt |
| HCT 42 | oncocytic follicular adenoma | −−− | na | wt | na | **m.10885delT** | AA61* | ND4 | + | **c.728delC, p.Ala159fs** | wt |
| HCT 43 | oncocytic follicular carcinoma | −−− | na | wt | na | **m.4222T>C** | S306P | ND1 | - | wt | wt |
| HCT 44 | oncocytic follicular adenoma | −−− | na | wt | na | **m.12797T>C** | L154P | ND5 | - | wt | wt |
| HCT 46 | oncocytic follicular carcinoma | −−− | na | wt | na | wt | wt | wt |  | wt | **NDUFB6; p.Glu8Val** |
| HCT 47 | oncocytic follicular carcinoma | −−− | na | wt | na | wt | wt | wt |  | wt | **NDUFA12 p.133del(Lys_Ile134insIle)** |
|  |  |  |  |  |  |  |  |  |  |  |  |
| a) nomenclature according to reference #7 | | |  |  |  |  |  |  |  |  |  |
| b) Description reported if specific features present | | |  |  |  |  |  |  |  |  |  |
| na=not availble | |  |  |  |  |  |  |  |  |  |  |

**Supplementary Table 2:** PROVEAN and SIFT output for the rare variants identified in oncocytic tumors.
