# Supplementary figures and images for: A mutation screening of oncogenes, tumor suppressor gene TP53 and nuclear encoded mitochondrial complex I genes in oncocytic thyroid tumors
Source: BMC Cancer. 2015 Mar 21;15:157. doi: 10.1186/s12885-015-1122-3 (PMC4374372; doi:10.1186/s12885-015-1122-3)

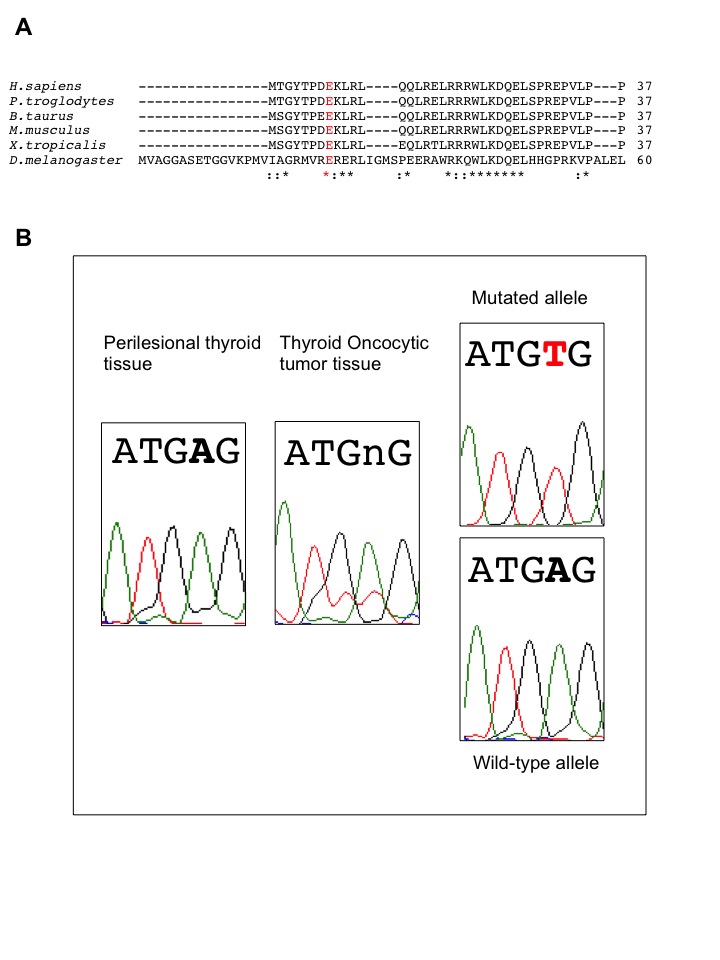

Supplement: Additional file 2: Figure S1. — (A) Protein sequence alignment showing the conservation across species of NDUFB6 p.8 Glu. (B) Electropherograms showing the novel missense change in NDFUB6 p.8 Glu > Val. Tumor tissue sample showing a somatic heterozygous profile, compared to perilesional tissue. The two different alleles were distinguished by cloning the PCR products into pcDNAII vector and sequencing the different clones. [file 12885_2015_1122_MOESM2_ESM.jpeg]
